# Reliable molecular identification of nine tropical whitefly species

**DOI:** 10.1002/ece3.1204

**Published:** 2014-09-11

**Authors:** Tatiana M Ovalle, Soroush Parsa, Maria P Hernández, Luis A Becerra Lopez-Lavalle

**Affiliations:** 1 Centro Internacional de Agricultura Tropical (CIAT)Km 17, Recta Cali-Palmira, Cali, Colombia; 2 CGIAR Research Program for Root Tubers and BananasLima, Peru

**Keywords:** COI, RFLP-PCR, Tropical whiteflies, Molecular identification

## Abstract

The identification of whitefly species in adult stage is problematic. Morphological differentiation of pupae is one of the better methods for determining identity of species, but it may vary depending on the host plant on which they develop which can lead to misidentifications and erroneous naming of new species. Polymerase chain reaction (PCR) fragment amplified from the mitochondrial cytochrome oxidase I (*COI*) gene is often used for mitochondrial haplotype identification that can be associated with specific species. Our objective was to compare morphometric traits against DNA barcode sequences to develop and implement a diagnostic molecular kit based on a RFLP-PCR method using the *COI* gene for the rapid identification of whiteflies. This study will allow for the rapid diagnosis of the diverse community of whiteflies attacking plants of economic interest in Colombia. It also provides access to the *COI* sequence that can be used to develop predator conservation techniques by establishing which predators have a trophic linkage with the focal whitefly pest species.

## Introduction

Whiteflies (Hemiptera: Aleyrodidae) are globally considered as one of the most important pests in agriculture (Bellotti and Arias^2001^; Morales and Anderson^2001^). They can attack a wide range of crop hosts often reducing their yields by more than 50% due to the combined effects of their feeding from phloem and their vectoring of plant viruses (Byrne and Bellows 1991). Most species of whiteflies are oligophagous or polyphagous; however, a few species are monophagous (feed on only one host plant; Resh and Cardé 2009). The most notorious whitefly species, *Bemisia tabaci*, can feed from over 500 different plant hosts worldwide (Greathead 1986).

Today, 1556 valid species are known to compose the Aleyrodidae family, the only included family in the Aleyrodidae subgroup of Hemiptera (Forero 2008). The Aleyrodidae is further subdivided into three subfamilies: Aleurodicinae, Aleyrodinae, and Udamoselinae (Forero 2008). The systematics of these subfamilies is based almost entirely on morphological characters of the forth-instar nymph (i.e., pupae), the most conspicuous developmental stage (Martin et al. 2000). Whitefly pupae, however, can exhibit significant phenotypic plasticity in response to differences in leaf architecture and to environmental or physical factors (Guershon and Gerling 2001), potentially confounding research outcomes. On the other hand, adult characters have been used successfully in the subfamily Aleurodicinae, but much work is still needed before they can be more broadly used in whitefly systematics (Ghahari et al. 2009). Modern molecular techniques can assist whitefly systematics research also facilitating species identifications (Oliveira et al. 2000; Calvert et al. 2001, 2005; Shatters et al. 2009).

Mitochondrial DNA (mtDNA) has been extensively used in phylogenetic studies of animals because it evolves much more rapidly than nuclear DNA, favoring the accumulation of nucleotide differences (i.e., polymorphisms) between closely related species (Brown et al. 1979; Lunt et al. 1996). The accumulation of these polymorphisms is primarily caused by the loss or gain of restriction sites without a detectable change in genome size (Hebert et al. 2003a).

The nature of the sequence changes within the mitochondrial cytochrome oxidase I (*COI*) gene make it an ideal candidate to be used as a DNA barcode system (Hebert et al. 2003b; Kress and Erickson 2008). Generally, sequencing of the polymerase chain reaction (PCR) fragment amplified from the *COI* gene is used for mitochondrial haplotype identification that can be then associated with specific species (Shatters et al. 2009). This approach can be particularly useful when rapid whitefly detection and identification is required for regulatory purposes especially when specimens are physically damaged. To be most valuable, the test needs to be highly accurate while maintaining high rates of throughput.

A basic approach to develop such type test has been to employ universal PCR primers to amplify the *COI* gene and to subject the products to restriction fragment length polymorphism (RFPL) analysis (Vidigal et al. 2002; Caldeira et al. 2003; Thyssen et al. 2005). In addition, a large number of species-specific *COI* sequences (nearly 150,000 species) have now been submitted to public sequence databases such as GenBank or The Barcode of Life Data System (BOLD) for use in taxonomic and phylogenetic studies of insects (Kwong et al. 2012; Ptaszyńska et al. 2012; Smith et al. 2012).

Our objective was to compare morphometric traits against DNA barcode sequences to develop and implement a diagnostic molecular kit based on a RFLP-PCR method using the *COI* gene for the rapid identification of whiteflies. This study will allow for the rapid diagnosis of the diverse community of whiteflies attacking plants of economic interest in Colombia. It will also provide access to the *COI* sequence that can be used to development predator conservation techniques. For instance, Lundgren et al. (2013) were able to establish which predators have a trophic linkage with *Aleurotrachelus socialis* in Colombia.

## Materials and Methods

### Morphological study

Over 40 years, CIAT's entomology research group has collected or received hundreds of whitefly voucher specimens from tropical and subtropical regions of the world, where beans, cassava, rice, tropical forages and fruits crops are grown by small holder farmers. These specimens deposited in the Arthropod Reference Collection at CIAT (CIAT-ARC) headquarters in Cali, Colombia constitute an invaluable resource for the identification and characterization of current, as well as potentially new tropical crop pest. Initially, pupae of whitefly were collected from multiple crops in the area of Palmira – Valle del Cauca (Table1). Pupae at the 4th instar nymph stage were morphologically examined under a light microscope and identified using the keys of Martin et al. (2000), Martin (2004), Hodges and Evans (2005), Martin (2005), and Dooley (Dooley 2006).

**Table 1:** List of whitefly species found associated with seven plant species in Palmira/Valle del Cauca (Colombia) based on morphological and anatomical features of the puparia

Host plant^1^	WF species identification^2^	Key used
*Citrus sinensis*	*Aleurothrixus floccosus*	Puparia dark brown to black; margin with coarse teeth, a gland is present at the base of each tooth, which gives margin the appearance of having a double row of teeth. Vasiform orifice elevated, small lingula obscured by operculum. Caudal furrow absent. Puparia often in dense groups which are covered by secreted flocculent wax (Martin 1987)
*Ipomoea indica*	*Aleurotrachelus trachoides*	Puparia black, marginal row of teeth much paler than the rest of pupal case, each tooth with an even paler glandular spot at its base. Inner submargin, mesad of marginal teeth and glands, with regular rows of coarse black. This dotted zone is divided into blocks by narrow breaks perpendicular to the margin; lingula large setose, expanded apically, protruding beyond vasiform orifice (Martin 1987)
*Manihot esculenta*	*Aleurotrachelus socialis*	Puparia dark usually black, elliptical, margin dentate, not differentiated at caudal or thoracic marginal openings. Glands present at the base of the marginal teeth giving the appearance of a double row of teeth. Small dorsal patches of tiny spinules, and rather stout eight abdominal setae that are longer than the caudal setae. Vasiform orifice subcircular to subchordate, longer than wide with an exserted lingula or it is obscured by operculum (Martin 2005)
*Manihot esculenta*	*Trialeurodes variabilis*	Body oval; submarginal papillae in an irregularly spaced single row; dorsal disc pores distad or interspersed among submarginal papillae; subdorsal tubercles absent; lingula head distinctly lobulated; first abdominal and eighth abdominal setae short (KeyLucid Central for Whitefly)^3^
*Matisia cordata*	*Lecanoideus floccissimus*	Submarginal zone without a ring of double-rimmed pores, only with a band of crowded wide-rimmed pores; dorsal disc mesad of compound pores only sparsely punctuated by septate pores (Martin et al. 2000)
*Passiflora edulis*	*Aleuronudus melzeri*	Cephalic and the three anterior abdominal compound pores subequal in size; 3 posterior abdominal compound pores reduced in size with the 5th posterior abdominal pore offset. Cephalic submedian setae absent. Lingula exerted (Cortez-Madrigal et al. 2008)
*Phaseolus vulgaris*	*Bemisia tabaci*	Puparia ovoid. Disc dorsal with or without setae. Caudal setae always stout, usually as long as the vasiform orifice whose sides are almost straight. Vasiform orifice triangular, inset from puparial margin by less than its own length. *Lingula spatulate* (Martin et al. 2000)
*Phaseolus vulgaris*	*Trialeurodes vaporariorum*	Body oval usually pale yellow; margin crenulations coarser, usually less than 13 occupying 0.1 mm; with a tiny tongue-like structure usually visible protruding beyond apical notch of vasiform orifice. Eight abdominal setae placed anterior to widest part of operculum. Legs lacking spines, only with setae (Martin et al. 2000)
*Psidium guajava*	*Aleurodicus dispersus*	Puparia relatively large (≥1.00 mm). Compound pores with cephalic pair similar in size to anterior most two abdominal pairs; each pore usually with a central process visible outer submarginal zone with a distinct ring of double-rimmed pores; dorsal disc mesad of compound pores densely punctuated by septate pores (Martin 2004; Martin et al. 2000)

1 WF infested plant materials were sampled at CIAT headquarters, Palmira, Colombia.

2 Determined by Maria del Pilar Hernandez (CIAT).

3 http://keys.lucidcentral.org/keys/v3/whitefly/Old/Homepage.htm.

Observations were carried out from January of 2011 to August of 2012 on CIAT's fields and laboratories located in Palmira, Valle del Cauca, Colombia. Pupae were collected from infested leaves of seven plant species and brought to the laboratory. Nine whitefly species were identified and 10–20 nymphs of each species (Table1).

The pupae samples were then placed vials and allowed to develop to adults. Once emerged, 10–20 adults per species were introduced in 1.5-mL microcentrifuge tube and placed in liquid nitrogen for DNA isolation.

### DNA isolation and PCR amplification

Total genomic DNA was obtained from nine whiteflies species using a modified cetyltrimethylammonium bromide method (CTAB; Doyle and Doyle 1990) by adding 2.5 M potassium acetate (pH 5.5) to precipitate protein. The DNA barcode region was amplified from all whitefly species using 10 *μ*M of universal DNA primers LCO1490 (5′-GGTCAACAAATCATAAAGATATTGG-3′) and HCO2198 (5′-TAAACTTCAGGGTGACCAAAAAATCA-3′) for the amplification of mitochondrial cytochrome c oxidase subunit I – *COI* (Folmer et al. 1994). PCR was conducted in a Mastercycler® pro (Eppendorf, Hamburg, Germany) for five cycles (40 sec at 94°C, 40 sec at 45°C, and 60 sec at 72°C) followed by 35 cycles (40 sec at 94°C, 40 sec at 51°C, and 60 sec at 72°C) with a final extension period of 72°C for 10 min. All PCRs were performed in a 12 *μ*L reaction volume using 0.25 U of Platinum® Taq (Invitrogen® Carlsbad, CA), 1× PCR Buffer (buffer composition), 2.5 mM of each dNTP (Promega Corp. Madison, WI), 2.5 mM MgCl_2_, and 20 ng of DNA.

### Sequencing of the *COI* region

Amplified *COI* region from each white fly species was cloned into the PGEM®-T Easy vector (Promega Corp.). Plasmid DNA from *Escherichia coli* used for sequence analysis was purified using a SV minipreps preparation (Promega Corp.). DNA inserts in this vector were sequenced in both directions using the Big Dye™ Terminator Cycle Sequencing kit with an Applied Biosystems 377 (Foster City, CA) DNA fragment analyzer by the Cornell University Life Sciences Core Laboratories Center (Ithaca, NY), using vector primers SP6 and T7. The resulting DNA sequences were edited and analyzed using Sequencher® 4.5 (Genes Codes Corp. Ann Arbor, MI).

### DNA sequence analysis

DNA Sequences were edited to the whitefly species-specific *COI* sequence by eliminating vector and universal primer sequence and analyzed for identity with sequences deposit in the GenBank, EMBL or BOLD public databases. *COI* sequence contigs were assembled from five clones per specie to identify unique haplotypes with a discrimination requirement of 100% identity. Haplotype nucleotide sequences of the whitefly samples are deposited in GenBank (Table2). Genetic distances were calculated with the alignment sequences for the nine species. Further, we used sequences reported in the GenBank for species listed in Table1 as control group, when available (*Trialeurodes vaporiariorum* and *B. tabaci*), and *Dialeurodes citri* (JQ340193), *Dialeurodes hongkongensis* (JQ340195) and *Dialeuropora* (JQ340197) as outgroup. Two phylogenetic reconstruction analyses were performed using the MEGA V6 and CLC main workbench V6.9 software. The first phylogeny analysis was performed using the statistical method neighbor-joining under Jukes-Cantor (J-C) Poisson-model for base substitution implemented in the CLC main workbench V 6.9 software (Jukes and Cantor 1969). The second analysis was also performed using the standard neighbor-joining (NJ) using the JTT matrix-based method (Jones et al. 1992).

**Table 2:** “In silico” characterization of the *COI* restriction sites using single enzyme reactions with *Alu*I and *Mbo*I or a double digest reaction with *Ase*I/*Taq*I in nine species of whiteflies

WF^1^ species	*Alu*I		*Mbo*I		*Ase*I + *Taq*I	
	No. frag.(s)	Length(s)	No. frag.(s)	Length(s)	No. frag.(s)	Length(s)
*Aleurodicus dispersus*	5	336, 174, 118, 69 & 12	2–4	615^2^, 611, 98, & 94	5–7	207, 172, 151, 95, 84 & 12^2^
*Aleuronudus melzeri*	4	330, 243, 124 & 12	7	328, 128, 78, 77, 53, 27 & 18	6	202, 172, 119, 95, 89 & 32
*Aleurothrixus floccosus*	3–5	Undigested (709)^2^, 591 536^2^, 118 & 55^2^	6	294, 128, 112, 104, 53 & 18	2	649 & 60
*Aleurotrachelus socialis*	3–6	611^2^, 454, 330^2^, 157 & 98	3–5	615^2^, 611, 53, 49^2^ & 45	3	554, 95 & 60
*Aleurotrachelus trachoides*	2–3	Undigested (709)^2^, 575 & 134	4	534, 77, 53 & 45	3	391, 263 & 55
*Bemisia tabaci* (A)^3^	3–7	591^2^, 454^2^, 336, 255, 118, 82^2^, & 36[Table-fn tf2-2]	5	483, 128, 71, 16 & 11	5	483, 95, 60, 50 & 21
*Bemisia tabaci* (B)^3^	4	367, 157, 98 & 87	6	534, 77, 53, 18, 16 & 11	5	354, 129, 95, 71 & 60
*Lecanoideus floccissimus*	5–7	522^2^, 454^2^, 336, 243^2^, 174, 118, 69 & 12	6–11	410^2^, 406, 132^2^, 128, 81^2^, 77, 53, 31^2^, 27 22^2^& 18	8	241, 133, 122, 95, 84, 21, 8 & 5
*Trialeurodes vaporiariorum*	1	Undigested (709)	5	456, 78, 77, 53 & 45	4	303, 174, 143 & 89
*Trialeurodes variabilis*	5–7	322^2^, 255^2^, 191, 134, 132, 131,121 & 47	6	212, 199, 134, 71, 66 & 27	5	255, 169, 130, 95 & 60

1 Whitefly.

2 Intraspecific variation explaining WF population differences.

3 Samples associated with *Phaseolus vulgaris*.

### Restriction mapping for rapid whitefly identification

Each of the 10 *COI* sequences obtained in this study was digested in silico with 30 commercially available restriction enzymes: eight base cutter *Not*I; six base cutters *Apa*I, *Ase*I, *Dra*I, *Eco*RI, *Eco*RV, *Hind*III, *Kpn*I, *Nde*I, *Pst*I, *Pvu*II, *Sac*I, *Sal*I, *Sca*I, *Sma*I, *Spe*I, *Sph*I, *Xba*I, and *Xho*I; five base cutters *Dde*I, *Hinf*I, and the four base cutters *Alu*I, *Hae*III, *Hha*I, *Hpa*II, *Mbo*I, *Mse*I, *Msp*I, *Rsa*I, and *Taq*I, using the “Restriction site analysis tool” of CLC main workbench 6.8.3 (CLC Bio, Aaurhus Denmark), to predict the product sizes of each *COI*-amplicon restriction reaction to implement a cost-effective *COI*-RFLP based species identification assay.

### *COI*-RFLP assay

Restriction endonuclease digestion of amplified *COI* fragments was performed with one unit of restriction endonucleases *Alu*I, *Mbo*I, and *Taq*I (NEB, Beverly, MA), 1× supplied restriction buffer and sterile Milli-Q H_2_O in a final volume of 20 *μ*L. Reactions were incubated at 37°C for 2 h. Restriction digestion products were resolved in 2% agarose gels in boric acid (BA) buffer at 80 V for 3 h, stained with GelRed™ (Biotium, Hayward, CA). Gel images were capture with a GelDoc™ BioRad documentation system and analyzed with the Image LAB™ software (BioRad, Hercules, CA).

## Results

### Molecular species identification (DNA barcoding)

Morphological characterization of the adult whitefly is very difficult and limits the capacity of identifying new species. Almost universally, whitefly taxonomical characterization is performed at the nymphal stage, which is labor intensive and time consuming. Because of the limitations for a rapid identification of whitefly species by conventional means, a molecular approach was adopted to assess its use for rapid identification of whitefly adult samples. Initially, the mtDNA *COI* universal primers LCO1490 and HCO2198 were employed to amplify the *COI* region of nine whitefly species. The *COI* gene (COX1) is one of 13 coding protein genes in the mitochondrial genome. It is ordinarily composed by 512 amino acids flanked on its 5′ terminus by tRNA-Trp-Tyr-Cys (15,121–15,315 bp) and at its 3′ terminus by tRNALeu (1538–1603 bp). The LCO1490 primer sequence is located 18 bp downstream near the 3′-end of the tRNACys, and the HCO2198 primer sequence is located on the opposite DNA strand 812 bp upstream at the 5′-end of the tRNALeu. Used together, these primers amplify a partial COX1 fragments commonly referred as the *COI* sequence or the Barcode of Life (Hajibabaei et al. 2007).

As identified morphologically, all nine whitefly species successfully amplified the *COI* sequence. The PCR amplification generated a fragment of approximately 709 bp that was subsequently cloned and sequence as described above. One hundred and fifty clones were recovered, but 54 were carrying the right size insert (approximately 5 per sample). DNA sequences obtained comprised 219 of the 512 amino acids that define the COX1 gene of the mitochondrial genome for all ten whitefly samples that represented the nine species under investigation (Table1). The sequences were employed to search the GenBank, EMBL, and BOLD databases to find novel sequences with the greatest identity. The information gathered from these three databases allowed the assignment of each whitefly sample to a single species (Table2).

**Figure 1:**
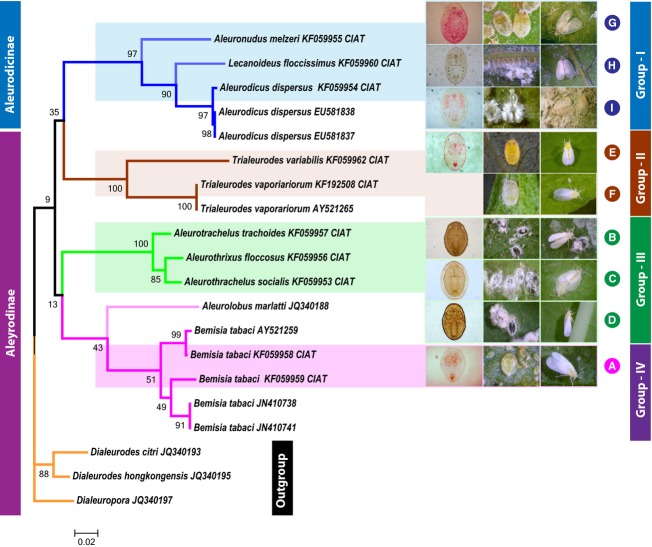
Phylogenetic relationships inferred using the neighbor-joining method in MEGA V6 and CLC main workbench V6.9 for tropical whitefly species, in relation to well-characterized whitefly taxa for which sequences were available in the GeneBank (Saitou and Nei 1987; Tamura et al. 2011). The optimal tree with the sum of branch length = 1.67299211 is shown. The percentage of replicate trees in which the associated taxa clustered together in the bootstrap test (10,000 replicates) are shown next to the branches (Felsenstein 1985). The analysis involved 29 amino acid sequences. There were a total of 219 positions in the final dataset. Four well-defined whitefly phylogenetic groups (G-I to G-IV) were observed. The nine species identified at CIAT were named from A to I. A =* Aleuronudus melzeri*, B = *Lecanoideus floccissimus*, C = *Aleurodicus dispersus*, D = *Trialeurodes variabilis*, E =* Trialeurodes vaporariorum*, F = *Aleurotrachelus trachoides*, G = *Aleurothrixus floccosus*, H = *A. trachoides*, and I = *Bemisia tabaci*.

DNA sequences of the partial *COI*region obtained in this study were subjected to phylogenetic analysis, along with seven related whitefly *COI*sequences retrieved from the GenBank/EMBL/BOLD databases, whereas three sequences *D. citri*(JQ340193), *D. hongkongensis* (JQ340195), and *Dialeuropora* spp. (JQ340197) were employed as out-group. All 20 *COI* DNA sequences were translated to amino acid sequences and a matrix with pairwise distance values was used to obtain a phylogenetic tree with neighbor-joining (Fig.1), and the agreed tree provided strong support for the morphologically determined whitefly classification (Table1). Trees obtained from MEGA and CLC main workbench gave essentially the same results. The *COI*amino acid sequence and phylogenetic analyses clustered all species in four groups. Group-I *Aleuronudus melzeriLecanoideus floccissimus*, and *Aleuronudus disperses,* group-II *Trialeurodes variabilis* and *T. vaporiariorum*, group-III clustered *Aleurotrachelus trachoides*. *A. socialisAleurotrachelus flocossus,* and in group-IV *B. tabaci* together with *Aleurotrachelus marlatti*. Of the four groups, group-I belonged to the subfamily Aleurodicinae, while groups II–IV including the outgroup belonged to the subfamily Aleyrodinae.

### *COI*–RFLP analysis

For the *COI*-RFLP analysis, *COI* fragments from the nine whitefly species were digested in silico with 30 restriction enzymes. Digestions with *Alu*I and *Mbo*I proved to be the most informative, whereas *Ase*I and *Dde*I are fully informative only if used together or in association with *Taq*I (Table2). The polymorphic restriction patterns revealed by *Alu*I or *Mbo*I in silico showed that for the 10 whitefly samples used in this study one can easily and inexpensively develop a diagnostic test for species identification.

**Figure 2:**
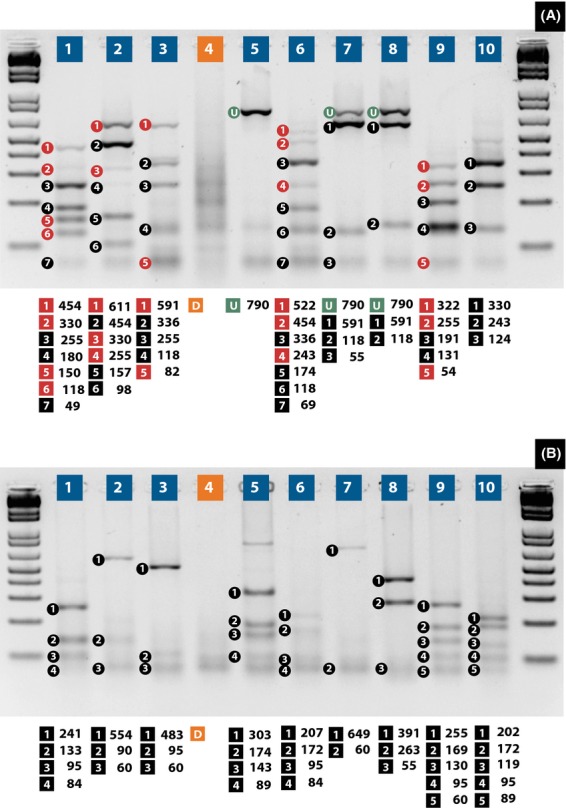
Displays two 2% agarose gels showing the restriction fragment length polymorphism profiles obtained by digesting the *COI* with *Alu*I (A) and *Ase*I + *Taq*I (B) enzyme from *Lecanoideus floccissimus* (lane 1)*, Aleurotrachelus socialis* (lane 2)*, Bemisia tabaci* (Sample 1, lane 3)*, B. tabaci* (Sample 2, lane 4)*, Trialeurodes vaporiariorum* (lane 5)*, Aleurodicus dispersus* (lane 6)*, Aleurothrixus floccosus* (lane 7)*, Aleurotrachelus trachoides* (lane 8)*, Trialeurodes variabilis* (lane 9)*, Aleuronudus melzeri* (lane 10). Molecular size markers are shown on the right and left side of the figure. Number filled in black corresponds to fragments size expected based on available sequence information and those in red to fragments expected by sequence variation due to intraspecies variation detected by RFLP-PCR of the *COI* amplicon.

The *COI*-PCR amplification yield both on genomic as well as cloned materials yielded fragments of 709 bp. After restriction enzyme digestion, all samples displayed reproducible restriction enzyme pattern. As predicted in silico, the profile produced by *Alu*I or *Mbo*I allowed us to distinguish among the nine whitefly species. *Alu*I showed the best profile for differentiating all ten samples (Fig.2A). *Mbo*I enzyme also allowed the identification of all samples; however, *A. trachoides* and *B. tabaci* DNA restriction profile where very similar (data not shown). The double enzyme identification system (*Ase*I/*Taq*I) also allowed distinguishing among all ten samples used here. The restriction patterns for some species were complex, and the sum of sizes of the bands produced upon digestion was not equal to the size of the undigested PCR products. For instance, in the patterns produced by *Alu*I for *L. floccissimus* (Fig.2A) the sum of the sizes of seven bands (454 + 330 + 255 + 180 + 150 + 118 + 49 bp) was not equal to the estimated 709 bp. It might be due to sequence variability of the *COI* gene caused by genetic differentiation among individual whiteflies originated from different populations. This is a very plausible explanation as DNA extractions were performed by randomly pooling the 10–20 adult whiteflies. However, the sensitivity for identifying genetic differences within the *COI* sequence disappeared when a double enzyme digestions were performed using *AseI *+ *TaqI*. In the patterns produced by these two enzymes for *B. tabaci* the sum of three bands (483 + 95 + 60) was not equal to the estimated 709. It might be due to overlapping of similar size fragments produced by the enzyme.

## Discussion

In the present study, we conducted classical morphological identification of the whitefly's 4th instar larvae and molecular identification of the adult form using the part of the COX1 gene region of the mtDNA. Based on the morphological traits, we identified nine species of whiteflies associated with seven crop species at CIAT's headquarters (Table1). This approach is limited because it demands specialized taxonomic expertise and it relies on a life-stage that may display significant environmentally induced morphological variation, confounding identifications (Neal and Bentz 1999).

To circumvent these limitations, we amplified Folmer's COX1 region (i.e., *COI*) to match DNA samples with morphologically identified whitefly species.

Before undertaken a phylogenetic inspection of the whitefly species sampled, *COI* sequences were further examined to characterize for the presence of orthologues or *COI*-like sequences by looking at the translated amino acid sequence. Moulton et al. (2010) suggested incorporating to the barcode methodology the analysis of the derived amino acid sequences particularly when a protein-coding gene region like *COI* is used. The translation of each *COI* sequence examined here consistently produced a fragment of 219 amino acids. Our aim to investigate the applicability of DNA barcoding for confirming species identifications of whiteflies was only limited when insufficient numbers of worldwide whitefly entries were available on GenBank and BOLD databases. This was predominantly the case for the South American region. Nevertheless, where whitefly *COI* sequence information was available, species identification was correctly and consistently achieved using GenBank or BOLD. Thus, DNA barcode identification was achieved for *B. tabaci* in cluster G-IV, *T. vaporiariorum* in cluster G-II, and *Aleurodicus dispersus* in G-I.

Our results suggest that *COI*sequence analysis is a very accurate and effective tool for species identification in whiteflies. Thus, by comparing molecular markers with diagnostic morphological traits, we provide six new *COI* sequences that will contribute to build a species-specific sequence library. A *COI*-based identification system will undoubtedly provide high resolution tools to nontaxonomist for conducting field surveys to collect information about the numbers and distribution of whitefly species among crops and its associated weeds.

*COI* sequence analysis has proven to be a good molecular marker for intra- and interspecific variation in whitefly species not only in this study but others (Thao and Baumann 2004; Boykin et al. 2007; Shoorcheh et al. 2008; Chu et al. 2012; Henri et al. 2013). However, a simple, rapid, and cost-effective identification system for whitefly species surveillance cannot rely exclusively on sequencing. Invariably in this study, the *COI* universal primes (Folmer et al. 1994) amplified a 709 bp fragment suggesting that a DNA diagnostic approach without a sequencing step will require either restriction enzyme digestion of the PCR product (RFLP-PCR) or develop molecular markers that adequately distinguish intra- and interspecific variation among different whitefly species. PCR amplification of the *COI* region followed by digestion with two single restriction endonucleases (*Alu*I or *Mbo*I) or a double enzyme system (*Ase*I + *Taq*I) could allow a rapid identification of whitefly species at the adult stage (Table2). This molecular method of identification can be easily used to monitor the spread of native whiteflies or for potential outbreaks of invasive exotic types in South America and elsewhere. As shown in Figure2(A and B), the *COI*-RFLP assay successfully differentiated all adult forms of the nine whitefly species identified morphologically at CIAT. We performed digestions with *Alu*I (Fig.2A) and *Ase*I + *Taq*I (Fig.2B) of the *COI* sequences obtained using total genomic DNA of bulked individuals for each whitefly species. The restriction profiles for all whitefly were screened using 2% agarose gels as they provide good resolution and size discrimination. These results demonstrate that *COI*-RFLP, using *Alu*I or *Ase*I + *Taq*I enzymes, is an important tool for differentiation of whitefly species and its biotypes. Furthermore, restriction digestions with the *Alu*I enzyme also allowed the identification of intraspecific changes indicating that this approach can also be used to conduct survey or monitor whitefly population distribution.

In addition to facilitating their authoritative identification, molecular protocols can also help characterize predator communities feeding on whiteflies (Lundgren et al. 2013). Molecular gut content analyses can establish which predators have a trophic linkage with a focal whitefly species, assisting the development of biological control programs for its management. Using this method, Lundgren et al.(2013) characterized the predator community of cassava whitefly *A. socialis* in Colombia. The *COI* sequence information generated in this study has the potential of facilitating similar efforts for a broader list of pestiferous whiteflies globally.

## Data accessibility

The COI sequence data used for this study can be found in the following links:

1. https://www.ncbi.nlm.nih.gov/nuccore/KF059953
2. https://www.ncbi.nlm.nih.gov/nuccore/KF059954
3. https://www.ncbi.nlm.nih.gov/nuccore/KF059955
4. https://www.ncbi.nlm.nih.gov/nuccore/KF059956
5. https://www.ncbi.nlm.nih.gov/nuccore/KF059957
6. https://www.ncbi.nlm.nih.gov/nuccore/KF059958
7. https://www.ncbi.nlm.nih.gov/nuccore/KF059959
8. https://www.ncbi.nlm.nih.gov/nuccore/KF059960
9. https://www.ncbi.nlm.nih.gov/nuccore/KF059962
10. https://www.ncbi.nlm.nih.gov/nuccore/KF192508
11. https://www.ncbi.nlm.nih.gov/nuccore/EU581837
12. https://www.ncbi.nlm.nih.gov/nuccore/EU581838
13. https://www.ncbi.nlm.nih.gov/nuccore/JQ340188
14. https://www.ncbi.nlm.nih.gov/nuccore/JQ340193
15. https://www.ncbi.nlm.nih.gov/nuccore/JQ340195
16. https://www.ncbi.nlm.nih.gov/nuccore/JQ340197
17. https://www.ncbi.nlm.nih.gov/nuccore/JN410738
18. https://www.ncbi.nlm.nih.gov/nuccore/JN410741
19. https://www.ncbi.nlm.nih.gov/nuccore/AY521259
